# Association between weight-adjusted waist index and cognitive impairment in Chinese older men: a 7-year longitudinal study

**DOI:** 10.3389/fnagi.2025.1510781

**Published:** 2025-01-21

**Authors:** Jie Lin, Hongchen Shen, Wenjuan Yang, Guojun Zhang, Jie Sun, Wenqin Shen, Li Huang, Huajian Chen

**Affiliations:** ^1^Guali Branch of the First People’s Hospital of Xiaoshan, Hangzhou, China; ^2^Renji College, Wenzhou Medical University, Wenzhou, China; ^3^School of Public Health, Wenzhou Medical University, Wenzhou, China

**Keywords:** weight-adjust waist index, cognitive impairment, older people, Chinese older men, dementia

## Abstract

**Background:**

Obesity, through mechanisms such as insulin resistance and systemic low-grade inflammation, can damage the central nervous system and impair cognitive function. Weight-adjusted waist index (WWI) is a novel measure of obesity that may offer more precise assessments of muscle and fat mass. This study aims to investigate the association between WWI and cognitive function in older Chinese men.

**Methods:**

Data from the 2011–2018 China Longitudinal Health and Longevity Survey (CLHLS) were used in this study. WWI and cognitive function were examined in both linear and non-linear situations using Kaplan–Meier survival curves, multivariate Cox regression models, and restricted cubic spline (RCS) regression.

**Results:**

This study included 1,392 older Chinese men aged 65 years and over for whom complete data were available. After controlling for all potential confounding variables, our analysis showed a statistically significant positive association between WWI and cognitive decline. Specifically, for every 1 cm/√kg increase in WWI, the risk of cognitive impairment increased by 17% (HR = 1.17, 95% CI: 1.02–1.35). Using 11.52 cm/√kg as the cutoff point for WWI, we found that High WWI was associated with a 44% increased risk of cognitive impairment compared with Low WWI (HR = 1.44, 95% CI: 1.07–1.96). RCS regression analysis confirmed a linear positive correlation between WWI and cognitive impairment.

**Conclusion:**

Higher WWI is linked to worse cognitive performance in older Chinese men.

## 1 Introduction

Dementia has emerged as one of the main global public health concerns as the world’s population ages (Livingston et al., 2017). Studies have shown that lower cognitive ability and significant cognitive decline continue to influence mortality in older people, even after controlling for other biomedical risk factors (Connors et al., 2015). Consequently, maintaining good cognitive performance has emerged as a crucial component of successful aging. The world’s highest population of dementia patients is found in China (9.5 million patients), followed by the United States (4.2 million patients) (Alzheimer’s Disease International, 2015).

Simultaneously, the incidence of obesity is increasing and has emerged as a serious global health concern. Studies have shown that obesity significantly increases the risk of adverse health outcomes in older people, not limited to cardiovascular disease and diabetes (Riaz et al., 2018; Piché et al., 2020), but also includes deleterious effects on the central nervous system and cognitive functioning (Mina et al., 2023; Miller and Spencer, 2014). Inflammatory factors produced by obesity will lead to disruption of cognitive functions mediated by regions such as the hippocampus, amygdala, and reward processing centers (Miller and Spencer, 2014). In addition, obesity, a feature of metabolic syndrome, has been identified as a risk factor for neurocognitive impairment (Anstey et al., 2011). Compared to normal waist circumference (WC), central obesity (high WC) has a higher risk of cognitive impairment and dementia, especially in older people. The body mass index (BMI) and waist circumference (WC) are commonly used to evaluate obesity in older people. It is noteworthy to acknowledge that these measurements are incapable of differentiating between adipose tissue and muscle mass.

The weight-adjusted waist index (WWI) is a new measure of obesity proposed by Park et al. The WWI integrates changes in body composition such as muscle and fat mass to provide a more comprehensive assessment of centripetal obesity (Kim et al., 2021; Park et al., 2018; Kim et al., 2023; Xu and Zhou, 2024). Previous studies have shown that WWI is negatively associated with appendicular lean mass and abdominal muscle mass among middle-aged and older people (Kim et al., 2021; Kim et al., 2022; Kim et al., 2023). Another study reported that WWI showed a stronger correlation with sarcopenic obesity in older men than other anthropometric measures such as waist-to-height ratio, BMI, and WC (Kim et al., 2023). And there was also an independent association between WWI and muscle-reducing obesity in men in specific populations (Tian et al., 2023).

Existing studies point to a correlation between obesity and cognitive impairment, but the relationship between WWI and cognitive function in Chinese older people has not been explored in the literature. Considering that obesity acts as a catalyst for disease and is a key cause of mortality in men (Holley-Mallo and Golden, 2021), we aimed to focus on the association between obesity and cognitive impairment in Chinese older men by investigating the correlation between WWI and cognitive performance in the Chinese older male population using 7-year longitudinal data obtained from the China Longitudinal Health and Longevity Survey (CLHLS).

## 2 Materials and methods

### 2.1 Study design and participants

The data were obtained from the China Longitudinal Health and Longevity Survey (CLHLS), conducted by Peking University. CLHLS was conducted in 23 provinces of China among people aged 65 and older and their adult children aged 35–64. This survey, which began in 1998 and was examined every 2–3 years, is the most extensive and longest-running social science survey in China. CLHLS systematically collects data on older people through face-to-face interviews conducted by trained staff. Details of the sample design have been described elsewhere and data quality was reported to be generally good (Zeng, 2012; Li et al., 2018; Liang et al., 2024).

**Figure 1:**
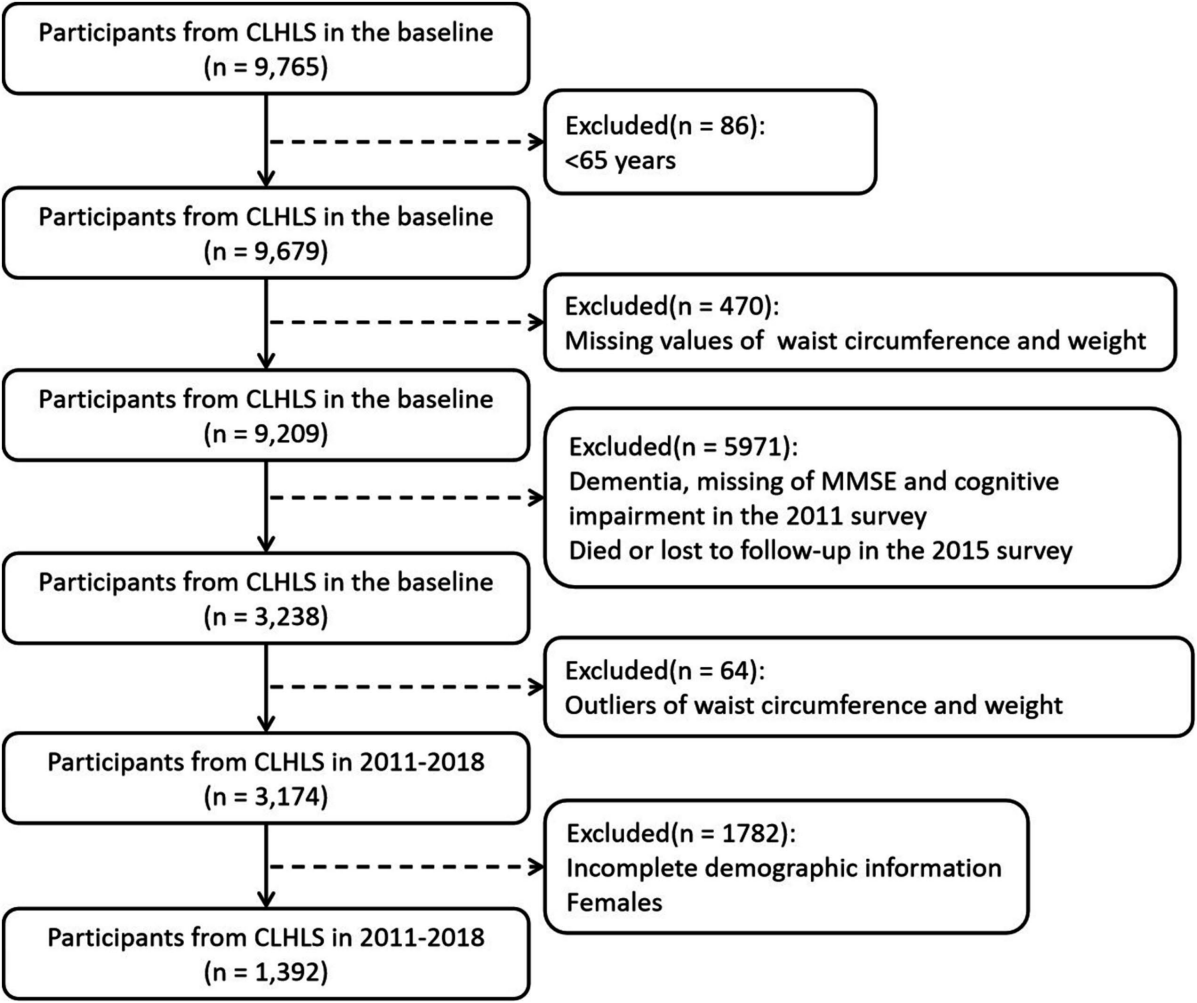
Flow diagram of how to select participants.

The study used 2011, 2014, and 2018 datasets of CLHLS. For this study, we selected participants aged ≥65 in 2011 as the baseline who completed the follow-up survey in 2014 and 2018. And participants with incomplete waist circumference and weight information were excluded. Participants who did not provide complete information on MMSE at 2011 and 2014 years were also excluded. Sample with cognitive impairment in 2011 and 2014 were excluded. In addition, in order to ensure that the time of cognitive impairment is complete, participants who died and were lost to follow-up were excluded in 2014. The sample selection for this study is shown in Figure 1. All participants signed written informed consent. And CLHLS was approved by the Research Ethics Committee of Duke University and Peking University (IRB00001052–13074).

### 2.2 Assessment of cognitive impairment

The Chinese version of the Brief Mental State Examination (MMSE) was used in the CLHLS to assess overall cognitive function (Yi and Vaupel, 2002). The MMSE consists of six dimensions with a total of 24 items (five items for orientation, three for registration, one for naming, five for attention and calculation, three for recall, and seven for language). MMSE scores range from 0 to 30, with higher scores indicating better cognitive function. The MMSE has been validated in Chinese older people population, with scores below 24 defined as cognitive impairment (Zhang et al., 2021).

### 2.3 Definition of weight-adjust waist index

The WWI was used as the independent variable and was calculated as the waist circumference (cm) divided by the square root of body weight (kg). Low WWI was defined as WWI < 11.25 cm/√kg while high WWI was defined as WWI ≥ 11.25 cm/√kg (Cai et al., 2022; Chen et al., 2024). For the choice of WWI cut-off value, a previous study in older people found that high WWI (≥ 11.25 cm/√kg) was associated with the highest all-cause mortality (Cai et al., 2022).

### 2.4 Covariates

To obtain more reliable results, we included factors that may be correlated with the dependent variable as covariates in the study. Based on the previous studies, 9 covariates from sociodemographic characteristics, lifestyle, and chronic diseases were considered. For sociodemographic characteristics, age, residence (urban, rural), marital status (married, other), occupation (professional work [skilled professionals, government, management], non-professional work [agriculture, fishing, services, industry, domestic work]), and education (no formal education [<1 year of education], formal education [≥1 year of education]), were included. Participants were defined as smokers/drinkers if current smokers/drinkers, regardless of frequency and quantity. Hypertension and diabetes mellitus were identified by participant self-report, with “yes” and “no” responses (Lv et al., 2019; Chen et al., 2024).

### 2.5 Statistical analysis

Categorical variables were expressed as counts (percentages) and continuous variables were expressed as means with standard deviations (SD). Differences in the occurrence of cognitive impairment between groups were detected using Kaplan–Meier (KM) survival curves and log-rank *P* tests. Hazard ratios (HRs) and 95% confidence intervals (95% CIs) were calculated by using Cox proportional hazards regression models to assess the association of WWI with cognitive impairment, and forest plots were drawn. The non-linear relationship between WWI and cognitive impairment was analyzed using restricted cubic spline (RCS) regression. All statistical analyses were performed using R (4.3.3) software. All *p*-values were two-tailed, with 0.05 being the threshold for statistical significance.

## 3 Results

### 3.1 Characteristics of the study population

The mean duration of follow-up was 4.4 years (standard deviation: 2.1 years), and 177 (12.7%) of the 1,392 participants developed cognitive impairment during this period. Participants had a WWI of 11 ± 1 cm/√kg. The results showed that participants with high WWI tended to be older, residence in the urban area, and other marital status compared to low WWI (Table 1).

**Table 1:** Baseline characteristics of the study population.

Variables	Overall (*n* = 1,392)	Low WWI (*n* = 814)	High WWI (*n* = 578)	*p*-value
Age, years	78.7 (8.3)	78.1 (8.3)	79.5 (8.3)	0.002
Education (%)				0.392
Formal education	1,040 (74.7)	615 (75.6)	425 (73.5)	
Informal education	352 (25.3)	199 (24.4)	153 (26.5)	
Occupation (%)				0.598
Non-professional work	1,167 (83.8)	686 (84.3)	481 (83.2)	
Professional work	225 (16.2)	128 (15.7)	97 (16.8)	
Residence (%)				0.022
Rural	684 (49.1)	421 (51.7)	263 (45.5)	
Urban	708 (50.9)	393 (48.3)	315 (54.5)	
Marital status (%)				<0.001
Married	962 (69.1)	591 (72.6)	371 (64.2)	
Other	430 (30.9)	223 (27.4)	207 (35.8)	
Smoker (%)				0.312
No	833 (59.8)	478 (58.7)	355 (61.4)	
Yes	559 (40.2)	336 (41.3)	223 (38.6)	
Drinker (%)				0.629
No	934 (67.1)	542 (66.6)	392 (67.8)	
Yes	458 (32.9)	272 (33.4)	186 (32.2)	
Hypertension (%)				0.196
No	994 (71.4)	592 (72.7)	402 (69.6)	
Yes	398 (28.6)	222 (27.3)	176 (30.4)	
Diabetes (%)				0.447
No	1,336 (96)	784 (96.3)	552 (95.5)	
Yes	56 (4)	30 (3.7)	26 (4.5)	
WWI, cm/√kg	11 ± 1	10.4 ± 0.7	11.9 ± 0.7	<0.001
Duration of follow-up, years	4.4 ± 2.1	4.5 ± 2	4.2 ± 2	
Cognitive impairment (%)				
Non-cognitive impairment	1,215 (87.3)	723 (88.8)	492 (85.1)	
Cognitive impairment	177 (12.7)	91 (11.2)	86 (14.9)	

### 3.2 KM survival analysis

**Figure 2:**
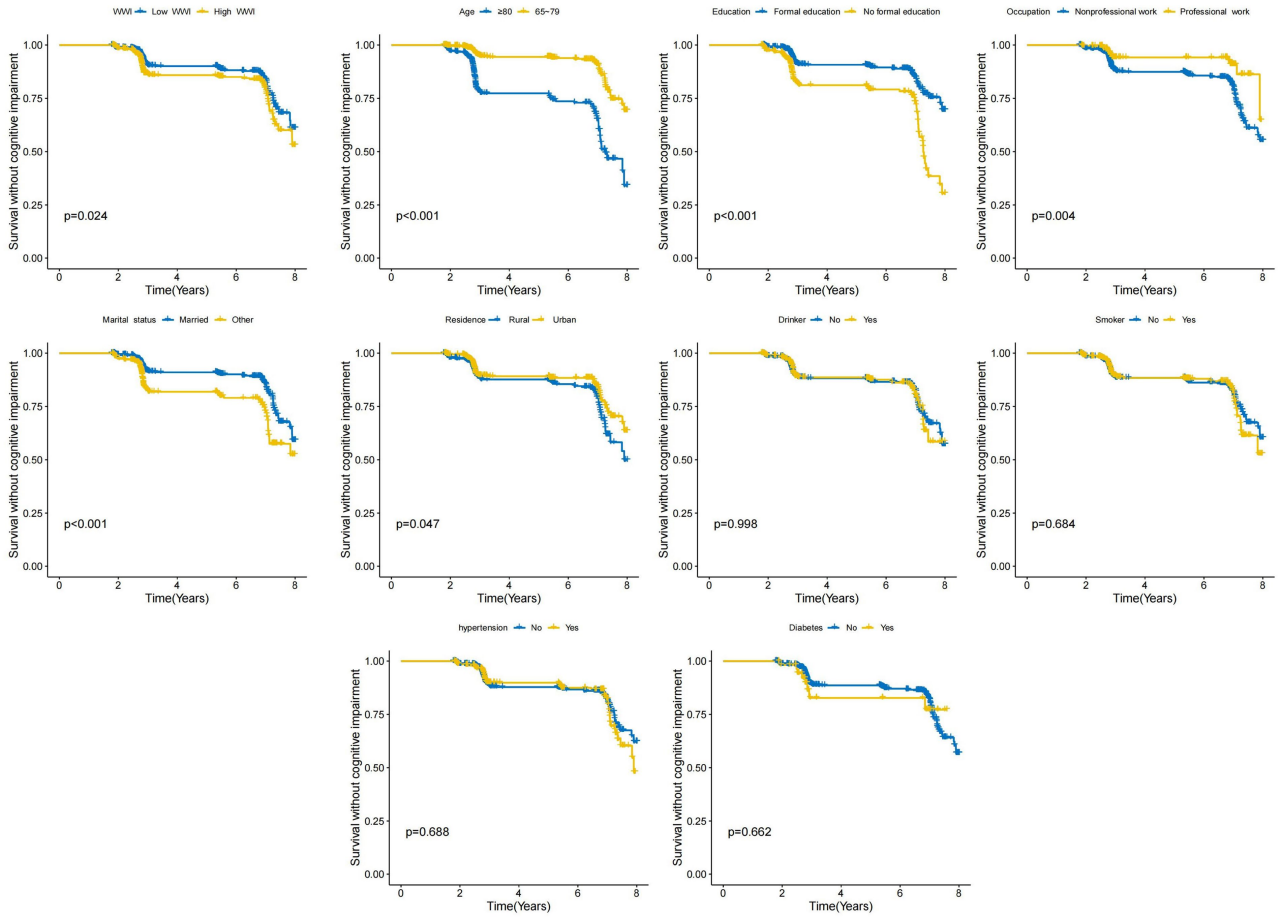
Kaplan–Meier (KM) survival curves for the association between characteristics of the study population and the risk of developing cognitive impairment were examined by log-rank *P*-tests for WWI, age, education, occupation, residence, marital status, smoker, drinker, hypertension and diabetes.

To explore exposure factors significantly associated with the development of cognitive impairment, we examined WWI, age, education, occupation, marital status, place of residence, drinker, smoker, hypertension, and diabetes by KM survival analysis and using log-rank *P* test, respectively, and visualized them using KM curves (Figure 2). We found that Low WWI, 65–79 years old, formally educated, professionally employed and married participants experienced lower cognitive impairment (All log-rank *p* < 0.05).

### 3.3 RCS and optimal thresholds

**Figure 3:**
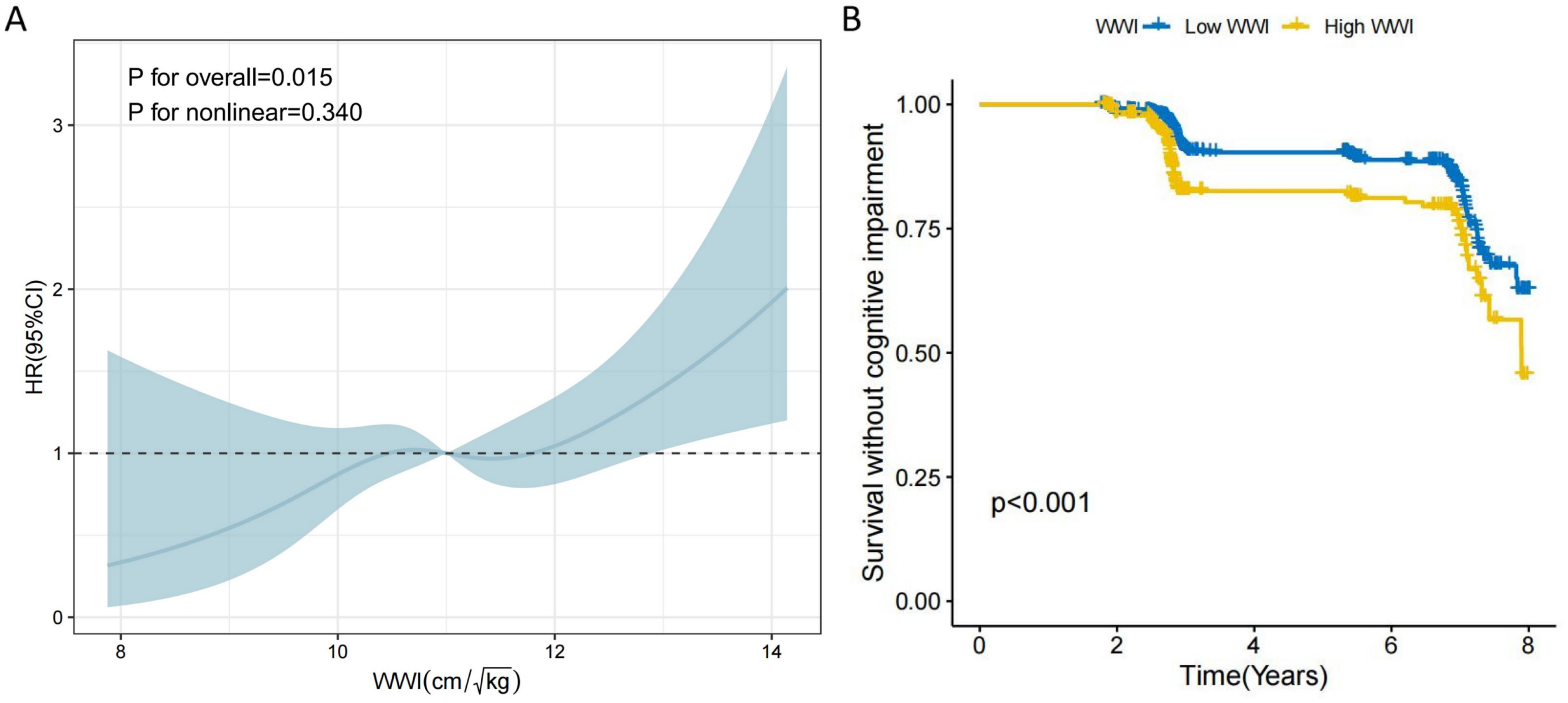
The RCS curve of WWI associated with the risk of developing cognitive impairment and the KM survival curve were the optimal thresholds. **(A)** The non-linear relationship between WWI and cognitive impairment was analyzed using a 4-section restricted cubic spline regression. **(B)** The optimal threshold of 11.52 was used as a cutoff point to distinguish between High WWI and Low WWI, and the difference in the risk of developing cognitive impairment between the two was detected using KM survival curve plotting as well as log-rank *P* test.

We further assessed the relationship between WWI and cognitive impairment using RCS analysis. The result showed a linear relationship between WWI and cognitive impairment (*P* for overall = 0.015, *P* for non-linearity = 0.407, Figure 3A). Immediately after, we used the ‘survminer’ package to discover that the optimal cutoff for WWI was 11.52 and Low WWI (<11.52) had a lower incidence of cognitive impairment (Figure 3B).

### 3.4 Correlation between WWI and cognitive impairment

Table 2 shows the relationship between WWI and cognitive impairment and observes a significant correlation between WWI and cognitive impairment. After adjusting for potential confounders (Model 3), for every 1 cm/√kg increase in WWI, there was an 17% increased risk of developing cognitive impairment (HR = 1.17, 95% CI: 1.02–1.35). Following the previous cutoff point (11.25), we found no significance between WWI and cognitive impairment after adjusting for potential confounders. However, through a cutoff of 11.52, we found that after controlling for potential confounders, older men with High WWI had a 44% increased risk of cognitive impairment compared with Low WWI (HR = 1.44, 95% CI: 1.07–1.96).

**Table 2:** Cox regression analysis of the relationship between WWI and cognitive impairment.

	Model 1		Model 2		Model 3	
	HR (95% CI)	*P*	HR (95% CI)	*P*	HR (95% CI)	*P*
WWI, cm/√kg	1.24 (1.08,1.43)	0.002	1.20 (1.05,1.38)	0.009	1.17 (1.02,1.35)	0.028
Low WWI (<11.25)	Ref	–	Ref	–	Ref	–
High WWI (≥ 11.25)	1.41 (1.05,1.89)	0.023	1.31 (0.97,1.75)	0.077	1.26 (0.94,1.69)	0.129
Optimal threshold						
Low WWI (<11.52)	Ref	–	Ref	–	Ref	–
High WWI (≥11.52)	1.72 (1.27,2.32)	<0.001	1.51 (1.12,2.05)	0.007	1.44 (1.07,1.96)	0.018

Model 1: no covariates were adjusted. Model 2: age was adjusted. Model 3: age, education, occupation, residence, marital status, smoker, drinker, hypertension and diabetes were adjusted. HR, hazard ratio; CI, confidence interval; WWI, weight-adjusted waist index.

### 3.5 Subgroup analysis

**Figure 4:**
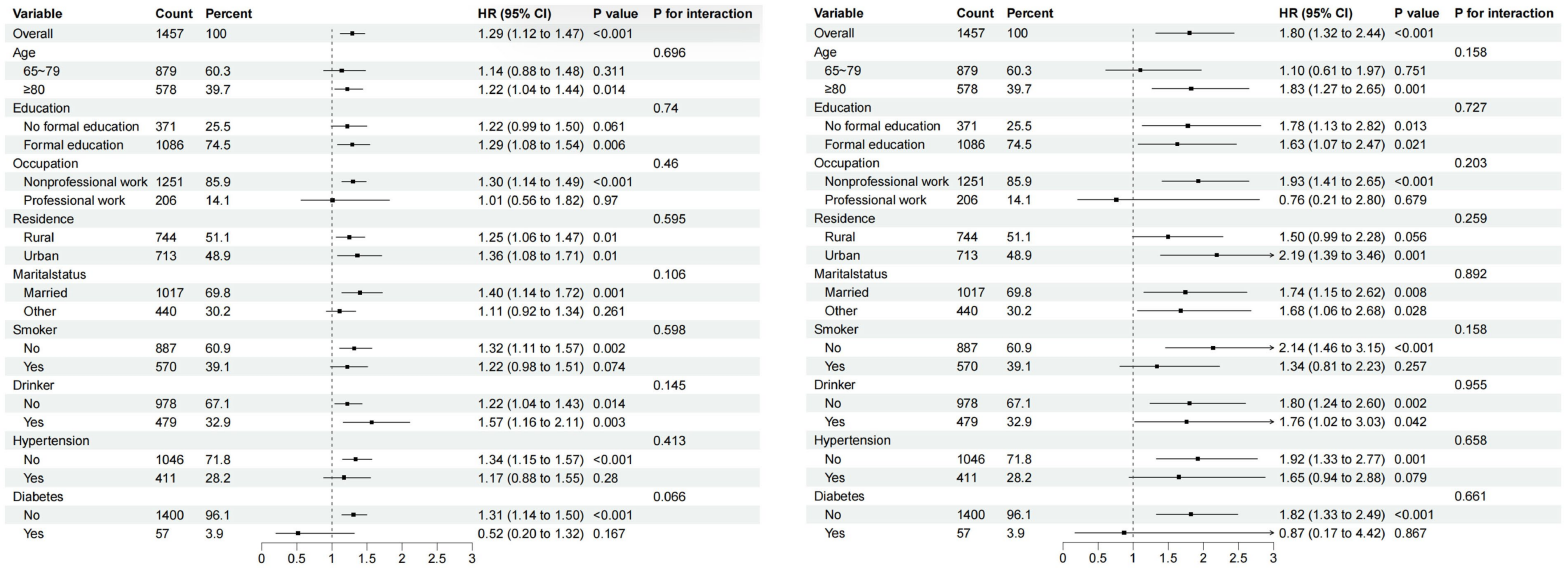
Subgroup analysis of the association between WWI and cognitive impairment. **(A)** Subgroup analysis of WWI (continuous variable). **(B)** Subgroup analysis of WWI (11.52 cm/√kg as a categorical variable at cutoff point).

We also performed subgroup analyses to explore whether the association between WWI and the risk of developing cognitive impairment remained stable across subgroups. As shown in Figure 4, both continuous and categorical variables (11.52 cm/√kg), all stratification variables including age (65–79 and ≥80 years), type of residence, marital status, education, occupation, smoking, alcohol consumption, diabetes mellitus, and hypertension did not influence the association between WWI and the risk of developing cognitive impairment (All *P* for interaction>0.05).

## 4 Discussion

This study aimed to investigate the relationship between body weight-adjusted waist index and cognitive function in a sample of 1,392 Chinese older men. The results of the study showed that higher WWI in older men was associated with an increased risk of cognitive impairment, and based on 11.52 cm/√kg as the cutoff point, high WWI had higher risk of cognitive impairment than low WWI. This finding suggested that WWI, a newly developed indicator of obesity, may be important in revealing the risk of cognitive impairment in older men. Whether WWI was considered a continuous or categorical variable, the association was consistently validated across subgroups, suggesting that higher levels of WWI are strongly associated with cognitive impairment in older men. The results of this study suggest that WWI may be a reliable indicator for assessing the relationship between obesity and cognitive impairment in older Chinese men.

To our understanding, this study is the first to assess the correlation between WWI and cognitive function in older Chinese men through longitudinal data, emphasizing the positive correlation between elevated WWI and cognitive decline. Existing research suggests that obesity has a negative impact on cognitive function. A prospective cohort study found a gradual decline in cognitive performance with increasing BMI, especially in individuals with a higher baseline BMI during the follow-up period (Cournot et al., 2006). Another cross-sectional study of 28,486 participants aged 60 years or older found that the prevalence of metabolic syndrome and its components (with the exception of hypertriglyceridemia), as well as the number of metabolic syndrome components, tended to increase as cognitive impairment worsened (MMSE scores decreased from ≥24 to 18–23 to 0–17) (Huang et al., 2022). In our study, the results of unadjusted and adjusted models provided evidence for WWI as an independent risk factor for cognitive impairment in Chinese older men. By using the optimal threshold for WWI to classify high and low WWI, we found that high WWI showed a higher risk of cognitive impairment compared to low WWI. RCS regression analyses further confirmed the positive linear correlation between WWI and cognitive decline.

Obesity is a state caused by an excessive accumulation of body fat. There is growing evidence that obesity has a significant negative impact on the central nervous system, specifically, obesity nearly doubles the risk of Alzheimer’s disease (AD) (Yu et al., 2020). The accumulation of visceral fat due to obesity and its associated metabolic consequences are closely linked to cognitive impairment, with these metabolic consequences forming the core features of metabolic syndrome, including insulin resistance, dyslipidemia, and hypertension (Kouvari et al., 2022). Systemic inflammation and insulin resistance have deleterious effects on the CNS and lead to cognitive impairment. Systemic inflammation is strongly associated with obesity. Pro-inflammatory cytokines (e.g., TNF-*α*, IL-1β, IL-6, MCP1) and inflammation-associated proteins (e.g., C-reactive protein, CRP) released from adipose tissue maintain the body in a state of low-grade systemic inflammation for long periods of time (Morys et al., 2021). This inflammatory state can further affect cognitive function and mood through dysregulation of the hypothalamic–pituitary–adrenal (HPA) axis (Dionysopoulou et al., 2021). Visceral obesity-induced insulin resistance severely affects optimal neuronal function, as evidenced by reduced capillary reactivity and cerebral blood flow (Heneka et al., 2015).

The WWI is an anthropometric measure used to assess increases in body fat mass and decreases in muscle mass. WWI is positively correlated with abdominal fat mass and negatively correlated with abdominal muscle mass (Kim et al., 2022). In contrast, the WWI offers a more accurate measure of total obesity because BMI is unable to distinguish between muscle mass and fat mass, and because WC has a strong correlation with BMI. A cross-sectional study including 10,289 Chinese hypertensive patients showed that WWI was an independent risk factor for dementia and was positively associated. This finding suggests that WWI can be a simple and reliable tool for assessing dementia risk (Zhou et al., 2023). Another study of 2,764 U.S. adults aged 60 and older similarly found that the higher the WWI, the worse the cognitive functioning (Huang et al., 2023). A study conducted by Levine and Crimmins (2012) showed that individuals aged 70 years and older with sarcopenia and obesity (either alone or in combination) had a significant decline in cognitive function compared to older people without sarcopenia or obesity. The subgroup analysis of this study did not find significant differences between subgroups in the association between WWI and cognitive function. This result suggested that the relationship between WWI and cognitive function is consistent across all subgroups. This consistency suggested that the WWI may be a stable and reliable indicator for assessing cognitive function in a variety of population backgrounds.

This study is notable for comprehensively analyzing the linear and non-linear relationships between WWI and cognitive function as assessed by the MMSE in a population of older Chinese men. This approach provides valuable insights into understanding the relationship between WWI and cognitive function and lays the groundwork for a multi-faceted study on the topic. In addition, our study included 1,457 nationally representative participants selected based on specific criteria. The adequate sample size allowed us to conduct subgroup analyses that allowed us to delve into the relationship between WWI and cognitive function in different populations. This approach enhances the statistical robustness and credibility of our findings. In particular, this was a 7-year cohort study, which allowed us to better explore the longitudinal relationship between WWI and cognitive performance. Compared to cross-sectional designs, cohort studies help to capture temporal sequentiality more accurately, thereby increasing the confidence that a longitudinal relationship may exist. However, there are some limitations to this study: (1) Due to the large number of variables involved that are related to WWI and cognitive performance, the results may have some inaccuracy even in a fully adjusted model, and we were unable to control for all potential confounders; (2) the categorization of educational level we used may not adequately capture the complex effects of education on cognitive functioning, and this categorization may limit the accuracy of the findings to some extent; (3) because diagnostic information for hypertension and diabetes is derived from individual self-reports, there may be a risk of recall bias, which in turn leads to misclassification, thus affecting the accuracy of the study results; (4) the exclusion of participants who died, failed follow-up, or had incomplete information resulted in a significant reduction in sample size, which may have affected the reliability of the study results; (5) the inclusion of only older men in this study, without considering the women, may have led to the omission of gender differences between obesity and cognitive functioning, thus affecting the generalizability of the results. Future studies should include women to more fully explore the mechanisms by which obesity affects cognitive function.

In this study, through the analysis of a 7-year longitudinal survey from the CLHLS, we identified high WWI as a significant risk exposure factor for cognitive impairment in elderly Chinese men, which predicts that WWI can become a novel indicator for assessing cognitive function in elderly Chinese men. Therefore, given the relative simplicity and ease of implementation of the WWI measurement, health practitioners can quickly obtain this indicator during a routine physical examination, thereby identifying individuals potentially at risk for cognitive decline at an earlier stage and taking timely and appropriate interventions to prevent further deterioration of cognitive function.

## Data Availability

The original contributions presented in the study are included in the article/supplementary material, further inquiries can be directed to the corresponding authors.
